# Urban Lake Health Assessment Based on the Synergistic Perspective of Water Environment and Social Service Functions

**DOI:** 10.1002/gch2.202400144

**Published:** 2024-09-03

**Authors:** Xueyuan Wang, Yuning Cheng

**Affiliations:** ^1^ Southeast University Xuanwu District Nanjing 210000 China

**Keywords:** social service function, synergistic development assessment model (SDAM), system health assessment, urban lakes, water environment

## Abstract

Urban lakes serve as vital ecological and recreational anchors within built environments, essential for enhancing urban resilience. Evaluating lake health predominantly focuses on water quality, assessing indicators such as nutrient levels, toxicity, pH balance, and water clarity to monitor changes. This study proposes a comprehensive evaluation framework that systematically describes specific spatiotemporal manifestations and periodic exogenous regulation characteristics across five dimensions: physical structure, water quality, shoreline dynamics, external regulation, and social service. Furthermore, it introduces an urban lake health assessment model based on synergistic development to evaluate the integrated development and interaction between water environments and social services. This model is applied across urban lakes in various developmental stages in China. Key findings include: 1) Urban development often impacts lake health disparately, with varying degrees of synergy observed between water environments and social services across different urban lakes. However, shifts in urban ideologies and improvements in governance, along with protective policies and project implementations, have contributed to improving water quality to some extent. 2) Engineering interventions do not consistently correspond with improvements in water quality, and governance measures sometimes yield mixed outcomes, underscoring the necessity for systematic solutions to lake health. Restoring hydrological processes emerges as crucial for enhancing sustainability.

## Graphical abstract

Urban lakes are crucial ecological and recreational assets within urban areas, significantly enhancing urban resilience. This study introduces a comprehensive evaluation framework that delineates the specific spatiotemporal manifestations and periodic exogenous regulations of lake water environments. Furthermore, it proposes a synergistic development assessment model to evaluate the integrated growth and interactions between water environments and social services.

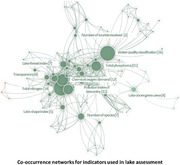

## 1 Introduction

Urban lakes are pivotal conduits of water, biological, and environmental resources, playing essential roles within the urban landscape. They form the cornerstone for maintaining urban ecological security and drive high‐quality environmental development in urban areas. Within the framework of China's ecological civilization, paradigms such as park cities and resilient cities underscore the growing importance of “systemic health” in urban habitat research.^[^ ^1^ ^]^ However, amid the dense development and intensive exploitation of environmental resources, urban lakes face challenges in maintaining their social service functions while experiencing an overall decline in water quality and ecological health.

Evaluation methods for urban lakes can be categorized into three main types. First, water quality assessment focuses on parameters such as nutrients, organic pollutants, heavy metals, chemical oxygen demand, dissolved oxygen, transparency, and turbidity,^[^ ^2^ ^]^ to provide precise water quality conditions,^[^ ^3^ ^]^ facilitating targeted interventions.^[^ ^4^ ^]^ Comprehensive indices such as the Water Quality Index,^[^ ^5^ ^]^ Trophic Level Index,^[^ ^6^ ^]^ and refined metrics systematically offer insights into eutrophication levels and overall water quality status, enabling comparisons across lakes over time.^[^ ^7^ ^]^ Second, biological assessments primarily utilize indices like the Benthic Macroinvertebrate Integrity Index^[^ ^8^ ^]^ to evaluate lake ecosystem health.^[^ ^9^ ^]^ Methods employing in situ biological assessments^[^ ^10^ ^]^ or biomarker techniques assess^[^ ^11^ ^]^ the composition of benthic macroinvertebrate communities, known for their effectiveness in ecological health evaluations.^[^ ^12^ ^]^ Third, comprehensive evaluations integrate multiple indicators to construct frameworks for assessing water environmental quality.^[^ ^13^ ^]^ Techniques such as fuzzy comprehensive evaluation,^[^ ^14^ ^]^ system dynamics modeling,^[^ ^15^ ^]^ artificial neural networks,^[^ ^16^ ^]^ and the Pressure‐State‐Response assessment method^[^ ^16^ ^]^ contribute to these frameworks. Statistical methods such as random forests and regression analyses identify significant factors influencing water environmental quality,^[^ ^17^ ^]^ including landscape patterns within lake basins,^[^ ^18^ ^]^ land use composition,^[^ ^19^ ^]^ water diversion,^[^ ^20^ ^]^ hydrodynamic influences,^[^ ^21^ ^]^ and topographical factors^[^ ^22^ ^]^ that intricately influence water quality dynamics.

Water quality monitoring plays a pivotal role in providing critical data at key moments and responding to environmental changes. However, relying solely on water quality assessments to evaluate the overall health and sustainability of lakes presents numerous limitations.^[^ ^23^ ^]^ These limitations are primarily evident in the transient nature of water quality assessments, their spatial constraints, and their ability to respond to long‐term changes in lake ecosystems.^[^ ^24^ ^]^ Water quality assessments often reflect the conditions of the moment they are taken, exhibiting significant dynamic variability. This implies that water quality can rapidly improve or deteriorate due to short‐term human interventions or natural fluctuations, but such changes may not necessarily be enduring. For instance, heavy rainfall can cause temporary declines in water quality, while management actions such as increased aeration or chemical treatments may rapidly enhance specific chemical indicators such as dissolved oxygen levels.^[^ ^25^ ^]^ However, these changes may not persist or accurately represent broader trends since they do not address the fundamental ecological structure or sources of pollution in the water body. Moreover, water quality monitoring typically relies on specific sampling points, which, despite being scientifically selected, may fail to comprehensively represent the entire lake's water quality. Different areas of a lake may exhibit significant variations in water quality due to differences in geography, hydrodynamics, and surrounding environmental conditions. For example, near‐shore areas may be more susceptible to terrestrial pollution compared to open water zones where water quality tends to be relatively better.^[^ ^26^ ^]^ Thus, while fixed‐point monitoring data are important, they offer limited insights into overall lake conditions.

Biological assessments serve as a core component in assessing the environmental status of urban lakes, providing comprehensive information about lake environmental quality over time and demonstrating advantages in sustainability. However, relying exclusively on biological assessments also has limitations, particularly in their application as factors for water environmental management.^[^ ^27^ ^]^ Biological assessments reflect long‐term and cumulative environmental conditions rather than immediate or transient changes. Biological indicators often exhibit time lags, meaning adverse environmental conditions may have persisted before anomalies in biological indicators are detected.^[^ ^28^ ^]^ In contrast, chemical or physical assessments offer more immediate insights into water quality parameters such as dissolved oxygen levels or heavy metal concentrations, which can be captured through real‐time monitoring systems to promptly adjust management strategies. Furthermore, while biological assessments offer critical insights into biodiversity and ecosystem health, translating these insights into specific environmental management actions can be challenging.^[^ ^29^ ^]^ For example, a decrease in a specific fish population may indicate issues within the food chain, habitat degradation, or declining water quality. However, identifying the precise influencing factors and designing effective intervention measures requires extensive investigation and robust data support.

The factors influencing urban lake water environments in Chinese cities are notably complex, characterized by substantial regional variability and profound external disturbances associated with the developmental stages of these urban centers.^[^ ^30^ ^]^ Throughout China's urbanization process, the complexity of issues surrounding lake water environments is particularly evident in the significant impact of urban development and land use changes on natural hydrological systems.^[^ ^31^ ^]^ First, the accelerated pace of urbanization has resulted in marked changes in land cover, particularly in the peripheries of lakes. Previously, these watershed areas were comprised of natural, highly permeable soft surfaces. However, with urban expansion, these areas have been increasingly replaced by impermeable hard surfaces. This transformation has substantially reduced natural groundwater infiltration and surface and soil runoff. Second, urbanization has altered the natural replenishment and drainage mechanisms of lakes.^[^ ^32^ ^]^ In their pristine state, lake levels and water volumes were primarily determined by factors such as precipitation, topographical features, and natural surface water collection, providing lakes with a certain degree of self‐regulation. However, urban development often disrupts original hydrological pathways and terrains, leading to the sealing off or redirection of natural runoff channels, thereby limiting the natural replenishment sources of lakes. Simultaneously, to meet urban drainage demands, artificial drainage systems are frequently directly connected to lakes, thus modifying the hydrological cycles and water quality conditions of these bodies of water. Furthermore, urban surfaces are predominantly covered with impermeable materials, which increase the transportation of various pollutants such as oils, heavy metals, and microplastics as early rainwater flows over urban surfaces.^[^ ^33^ ^]^ Ultimately, these pollutants find their way into lakes, thereby affecting their water quality and ecosystem health. Presently, many urban lakes in China depend heavily on extensive external water diversion and regular dredging projects to maintain their aquatic ecosystems. Notable urban lakes like Xuanwu Lake in Nanjing, Jinji Lake in Suzhou, and West Lake in Hangzhou each regulate their water quality through daily average water diversions of 200 000, 800 000, and 300 000 tons, respectively. An analysis spanning over eight years on Chinese lake water quality has revealed that variations in water supply significantly influence the WQI and TSI of lakes.^[^ ^34^ ^]^ Urban lakes in China generally exhibit characteristics such as eutrophication and ecological fragility. Changes in land use patterns have not only altered the overall water environment of lake watersheds but have also contributed to the degradation of lake ecological processes and landscapes, thereby substantially increasing the costs and challenges associated with urban construction and management operations.

The multi‐index comprehensive evaluation method encompasses various scales and ecological groups, establishing a close and systematic relationship with environmental factors. These evaluation indices serve as effective leverage for enhancing the health status of lakes. In recent years, research has increasingly focused on the synergy between urban development and urban water environments,^[^ ^35^ ^]^ becoming a crucial approach to optimizing urban‐water relations. The interpretation of systemic health in lake water environments forms the basis for establishing evaluation criteria. Currently, scholars emphasize different aspects of defining lake health. The mainstream viewpoints can be categorized as follows: One perspective asserts that a healthy lake demonstrates sustainability and stability, maintaining its organizational structure over time and capable of automatic recovery after disturbances.^[^ ^36^ ^]^ Another viewpoint suggests that lake system health encompasses meeting reasonable societal demands and the lake ecosystem's ability for self‐sustenance and renewal.^[^ ^37^ ^]^ Systemic health requires lakes to maintain the integrity of material cycles and energy flows even under damage,^[^ ^38^ ^]^ thereby presenting overall diversity and complexity.^[^ ^39^ ^]^ Based on these perspectives, this study defines urban lake system health as the capacity of lakes to meet environmental quality and societal service requirements. Simultaneously, the internal structure and organization of lake ecosystems exhibit stability and metabolic capabilities, demonstrating resilience to external pressures. Following sudden natural or anthropogenic disturbances, lakes can maintain essential functions and structures.

Drawing from foundational research and current contexts, this study endeavors to construct a method for assessing water environmental quality that comprehensively captures the specific temporal, spatial, and periodic disturbance characteristics of lakes. Additionally, it integrates a synergistic development evaluation approach into lake health assessment, establishing a model that evaluates the co‐evolution of water ecological environmental quality and social service functions. This methodology not only provides a multifaceted reflection of the varying levels and conditions of lake system health through multiple indicators but also enhances understanding of the reciprocal interactions between social development and surface water systems. It is instrumental in identifying strategies to enhance the health of lake ecosystems.

## 2 Method and Data

### 2.1 Systematic Evaluation of Water Environment Quality and Social Service Function of Urban Lakes

**Figure 1:**
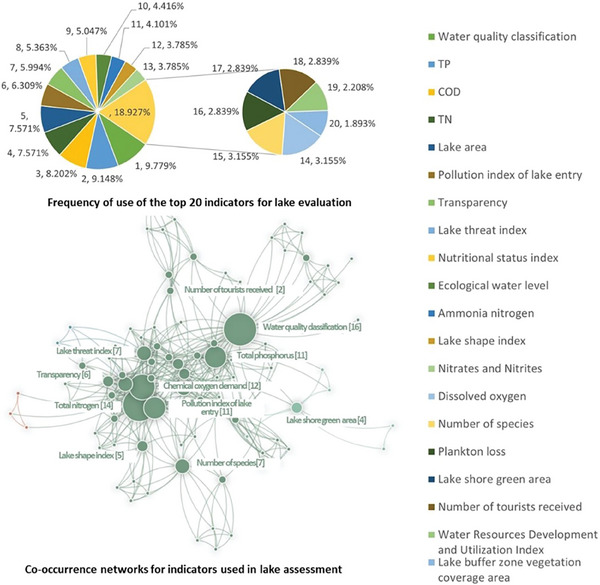
Analysis of the frequency of use and co‐occurrence network of lake evaluation indicators.

Urban lakes constitute complex ecosystems shaped by extensive natural changes and human socio‐economic activities. In the context of assessing urban lake system health, this research examines both water environmental quality and social service functionalities. Employing the analytic hierarchy process, it constructs a hierarchical evaluation framework encompassing subsystem, criterion, and indicator levels. Drawing from a comprehensive review of authoritative and impactful literature, spanning the period from 2015 to 2024 and sourced from the Web of Science database, a total of 274 pertinent articles on lake health assessment were identified. Through systematic analysis, 42 key indicators were extracted and organized into five distinct categories: lake physical structure, lake water quality characteristics, shoreline characteristics, external regulation characteristics, and societal service characteristics. These classifications were derived based on the intrinsic meanings of the indicators and their interrelationships. The study categorizes the index layer into jointly selected features and specific indices. Jointly selected features denote specific objective attributes of the lakes under study, while specific indices delineate differences in the structure, environment, and functionality of urban lakes. Through an assessment of usage frequency and categorization, key indicators demonstrating robust applicability and high represent were compiled, as depicted in **Table** **1**. Analysis of the top 20 indicators by frequency of use and their co‐occurrence network is illustrated in **Figure** **1**.

**Table 1:** Co‐selected characteristics and specific indicators for lake evaluation generalized from journal articles.

Criteria Layer	Co‐selected features	Specific indicators	Reason for indicators selection
Lake Physical Structure	Lake area characteristics Lake morphological characteristics Lake vertical characteristics	Lake area	Key indicators that affect lake water temperature stratification, dissolved oxygen distribution, and biological habitat.
		Lake fractal dimension	The higher the fractal dimension, the more complex the lake morphology, which usually means a higher diversity of biological habitats and complexity of the ecosystem.
		Lake shape index	The shape index reflects the geometric characteristics and regularity of a lake, affecting its water flow, sediment distribution, and ecological processes.
		Shoreline development factor	It refers to the ratio of the tortuosity of a lake's shoreline relative to its area. A high edge development coefficient means that the lake has more shoreline microhabitats, which are suitable for a variety of aquatic plants and animals.
		Lake near‐circularity	It is used to describe the degree of closeness of the shape of a lake to a perfect circle, calculated as the ratio of the actual lake area to the area of an ideal circle with the same circumference. Lakes with a lower degree of closeness to a circle have more complex shapes, more bays and protrusions, and provide a variety of ecological environments.
		Lake depth	Lake depth affects the temperature stratification, dissolved oxygen distribution, and biological habitat of the water body, and is an important factor in the structure and function of the lake ecosystem.
		Ecological water level	Reflects the basic water demand of lakes in maintaining ecological functions and biodiversity.
Lake water quality	Eutrophic characteristics Characteristics of oxygen‐consuming organic matter Dissolved oxygen characteristics	Total nitrogen (TN)	Reflects the total nitrogen content in the lake; high values may indicate excessive nutrient input, triggering ecological problems such as algal blooms.
		Total phosphorus (TP)	Indicates the accumulation level of phosphorus, a key factor affecting algal growth, and too high can lead to eutrophication.
		Water quality classification	The system evaluates the water quality of lakes and classifies water bodies according to specific standards, which directly reflects the ecological health.
		Nutritional status index	It measures the nutrient level and eutrophication degree of lakes. High values indicate that the lake may be experiencing ecological degradation.
		Plankton loss	Refers to the reduction in the number of plankton and can be used to assess water pollution and ecosystem stress.
		Pollution index of lake entry	It reflects the pollutant load flowing into the lake and is an important indicator for assessing external pollution pressure.
		Hydrogen Potential (pH)	Reflecting the acidity and alkalinity of water bodies, deviations in pH values indicate that water quality may be affected, affecting the survival of aquatic organisms.
		Transparency	A measure of the clarity of a body of water, reduced clarity is often associated with increased suspended matter and deteriorating water quality.
		Permanganate Index	It indicates the content of organic matter and easily oxidized substances. A high value indicates that there are more pollutants in the water.
		Chemical Oxygen demand (COD_Mn_)	Measures the amount of oxygen‐demanding chemicals in water and reflects the degree of water pollution.
		Chlorophyll a	It mainly reflects the algal biomass and its high content is usually associated with the eutrophication of water bodies.
		Diatom ratio	The amount of diatoms reflects the water quality. Generally, the water ecology with a high proportion of diatoms is healthier.
		Total biomass	Measures the total amount of organisms in a lake, reflecting the productivity and health of the ecosystem.
		Number of species	An indicator of biodiversity, high species richness generally indicates healthier ecosystems.
		Dissolved Oxygen (DO)	It reflects the balance of biological activity and redox processes and directly affects aquatic organisms, as well as water quality stability and eutrophication.
		Ammonia nitrogen	Refers to the ammonia content in water. High ammonia nitrogen levels can cause water quality deterioration and be toxic to aquatic life.
		Nitrates and Nitrites	Reflects the state of the nitrogen cycle; abnormal levels may indicate pollution or ecological imbalance.
Lake shoreland characteristics	Lake shore features Land use characteristics of the basin	Lake shore green area	Indicates the area of green space around the lake, which helps provide habitat, enhance biodiversity, and provide natural solutions to the urban heat island effect and sewage treatment.
		Lake buffer zone vegetation coverage area	The buffer zone is the transition zone between the lake and the surrounding land, and its vegetation coverage is critical for preventing soil erosion, absorbing pollutants, and maintaining the ecological balance of the water body.
		Dominant plant coverage area	Describes the total area covered by the dominant plant species in the lake's shore zone, reflecting the stability of the ecosystem and the health of the biological community.
		Cultivated land occupation rate	It reflects the proportion of cultivated land in the watershed, which directly affects the nutrient load of the lake, such as the input of nutrients such as nitrogen and phosphorus, which may lead to water quality problems such as eutrophication.
		Slope structure type	Describe the geological and soil structure of lake slopes, including slope steepness, soil type, and stability, which have an important influence on lake erosion and deposition processes.
		Shallow water plant coverage	Indicates the coverage of aquatic plants in shallow water areas. High coverage helps water purification and maintain biodiversity.
		Number of dams per kilometer	Reflecting the density and intensity of human intervention, the number of high levees may weaken the ecological functions of natural coastlines.
Exogenous regulation characteristics	Environmental governance characteristics Land use characteristics of the basin	Environmental Governance Investment Index	Shows the financial investment in lake environmental protection and governance. A higher investment index is generally associated with improved lake health.
		Annual water replenishment scale	This metric describes the total amount of water that is replenished in a lake in a year. This helps assess how resilient a lake is to drought and water level declines, and whether it can continue to provide needed ecological services.
		Desilting depth	It refers to the depth of clearing sediments from the lake bottom. Appropriate dredging depth can improve water quality and enhance the ecological function of the lake bottom.
		Lake flow variation index	This indicator is used to measure the degree of variability in the amount of water flowing into the lake. A high variability index may indicate unstable water flow, which will have an impact on the lake's ecosystem, such as affecting the reproduction cycle and living environment of aquatic organisms.
		Lake Threat Index	Quantify the ecological pressure on the lake caused by activities such as fishing, hunting, mowing, and reclamation. A lower threat index means less human interference.
Social service characteristics	Sightseeing tourism features Water resource utilization characteristics Flood storage characteristics	Number of tourists received	Record the number of tourists a lake receives within a certain period of time. A high number of visitors indicates that the lake has high attractiveness and public value.
		Attention	Indicates the degree of public attention to lakes, including media coverage, social media discussions, etc. High attention is often closely related to public participation and policy makers' attention, affecting the protection and utilization strategies of lakes.
		Public Satisfaction Index	Reflecting the public's overall satisfaction with urban lakes, high satisfaction generally indicates good lake environmental quality and accessibility.
		Water Resources Development and Utilization Index	Reflects the extent and efficiency of the development and utilization of lake water resources. This includes drinking water supply, agricultural irrigation, industrial water use, etc. Efficient water resource utilization indicates that the management strategy of the lake can meet multiple needs while protecting the health of the water body.
		Water functional area compliance index	It shows the extent to which each water functional area of the lake meets the preset environmental protection standards. The higher the index, the better the water quality and functional area management.
		Cultural value of scenic spots and historical sites	Assess the historical and cultural value of the lake and its surrounding areas. High cultural value enhances the social and educational functions of the lake.

This study aims to develop a system‐based assessment framework for urban lake water environmental quality, focusing on ecosystem health. The framework comprehensively captures two main aspects: specific spatiotemporal manifestations and periodic exogenous regulations. Spatiotemporal manifestations include the physical structure, water quality, and shoreline characteristics of lakes at specific times or developmental stages, closely linked to urban development phases. Exogenous regulation, such as water diversion, supplementation, and sediment dredging, exhibits distinct periodicity and anthropogenic control characteristics, typically relying on policy directives and urban planning in Chinese cities as proactive interventions.

Specific spatiotemporal manifestations reflect key aspects such as water quality, physical structure, and water ecosystems of lakes. Meanwhile, characteristics of exogenous regulation closely relate to lakes' adaptive capacities against disturbances and changes. These quantified representations are pivotal in assessing overall lake ecosystem health and stability, forming criteria for evaluating lake water environmental quality. This framework facilitates dynamic assessment and comprehension of lakes' responses to natural conditions and urban activities, thereby revealing transient changes and long‐term trends in ecological and environmental conditions. Based on selected lakes and assessment scenarios, considering water system status, data availability, and validity, jointly selected features and specific indicators are determined to construct the indicator system framework (**Table** **2**). Principles followed in indicator selection include:

1. Principle of Independence: To avoid redundancy, indicators should be as mutually independent as possible.
2. Principle of Quantitative and Qualitative Integration: Indicators should be easily measurable and statistically relevant to local data and time‐series observations from designated monitoring stations. Descriptions of lake system health inevitably include qualitative aspects; if critical indicators are challenging to quantify, they are characterized through appropriate qualitative assessments.
3. Principle of Operability: While ensuring a comprehensive reflection of complex lake ecosystems, data required for the indicator system should be relatively accessible and based on practical statistics, making the system widely applicable and readily accepted.

**Table 2:** Health evaluation system of water environment quality and social service function of urban lakes.

Subsystem level	Weights	Criterion level	Indicator level	Weights	Indicator Direction	Data sources and calculation methods	Unit
Quality of the water environment of urban lakes	0.6978	Characterization of the physical structure of lakes	Lake area	0.0269226	+	Lake survey report statistics	km^2^
			Lake depth	0.0319239	+	Lake survey report statistics	m
			Lake shape index	0.0538776	+	$\frac{\mathrm{area}}{\mathrm{longest}\;\mathrm{axi}s^{2}}$ (Reflects the geometric characteristics and regularity of the lake)	–
			Shoreline development index	0.0622341	+	$\frac{\mathrm{round}}{\sqrt[{2}]{\pi *\mathrm{area}}}$ (Reflects the diversity of habitat conditions along lake shores)	–
			Lake near‐circularity	0.0646308	+	$\frac{4\pi \;*\;\mathrm{area}}{\mathrm{roun}d^{2}}$ (Reflects the bay and prominence of the lake)	–
		Lake water quality	Water quality classification	0.0874783	–	Local ecological bulletin statistics	–
			COD_Mn_	0.0838746	–	Local ecological bulletin statistics	mg L^−1^
			TP	0.0795051	–	Local ecological bulletin statistics	mg L^−1^
			TN	0.0732033	–	Local ecological bulletin statistics	mg L^−1^
			DO	0.1289097	+	Local ecological bulletin statistics	mg L^−1^
			Transparency	0.0580698	+	Local ecological bulletin statistics	m
		Characterization of lake shoreline zones	Shoreland green space to lake area ratio	0.0595971	+	GIS‐based statistical calculations	–
			Percentage of vegetation cover in the 60‐meter buffer zone	0.0657909	+	GIS‐based statistical calculations	–
		Exogenous regulation characteristics of lakes	Amount of recharge water	0.0661671	–	EPA Publicly Available Statistics	ton year^−1^
			Dredging depth	0.0578151	–	EPA Publicly Available Statistics	cm
Social service functions of urban lakes	0.3021	Number of tourists visits		0.63785185	+	MGTO public statistics	million visitors
		Concerned about the heat		0.15320175	+	Baidu Search Frequency Statistics	million visits
		Public satisfaction index		0.2089464	+	MGTO public statistics	–

Lakes exhibit a 3D physical structure encompassing parameters including surface area, depth, shoreline development, lake perimeter curvature, and morphological indices. Horizontally, lakes feature a borderline of land‐water interfaces where terrestrial ecosystems influence the upper edge and aquatic systems influence the lower edge, affected by processes like water flow erosion.^[^ ^40^ ^]^ The diversity of shoreline morphology determines variations in lake depth, creating habitats for diverse plant communities adapted to specific water conditions.^[^ ^41^ ^]^ Lake morphology and shoreline features also dictate the diversity of water flow rates and dynamic changes within lake bodies.^[^ ^42^ ^]^ The morphological characteristics of lakes are crucial for nutrient cycling and hydrodynamic processes within lake ecosystems, influencing hydrological conditions, biodiversity, and material and energy circulation.^[^ ^43^ ^]^ Studies have indicated correlations between lake morphology, evolutionary patterns of lakes, and erosion dynamics of elastic shore zones.^[^ ^44^ ^]^ The richness of lake morphology and structural stability impact water depth and flow velocities, and guide hydrodynamic characteristics within lakes. Lake water quality indicators include lake water classification, Chemical Oxygen Demand (COD_Mn_), Total Nitrogen (TN), Total Phosphorus (TP), Dissolved Oxygen (DO), and transparency. Water quality classification reflects overall water quality based on a range of indicators including nutrient status, toxicity characteristics, and pH balance, systematically indicating suitability for various uses, potential ecological risks, and management needs. TN and TP are critical indicators for assessing nutrient status, directly influencing eutrophication levels in lakes. COD_Mn_ measures the total amount of organic and some inorganic substances that can be chemically oxidized in water, indicating pollution levels. Dissolved oxygen levels in lakes directly indicate the survival conditions for aquatic organisms, affecting their metabolism and well‐being. Transparency reflects the concentration of suspended particles and the depth to which light penetrates the water. Lower transparency often signifies higher suspended solids, which may originate from external sediment input or internal algal proliferation. The indicators of lake shoreline zones encompass metrics such as the ratio of shoreline green space to lake area and the vegetation coverage within the 60‐meter buffer zone. As critical transition zones between lakes and terrestrial environments, shoreline ecosystems play pivotal roles in purifying water quality, providing habitats for biodiversity, and facilitating the exchange of materials and energy between land and water.^[^ ^45^ ^]^ The ratio of shoreline green space to lake area reflects the relationship between ecological conservation areas at the lake edge and the overall lake size. A higher ratio generally signifies healthier ecological margins capable of supporting diverse habitats, maintaining biodiversity, and enhancing pollutant filtration. Elevated vegetation coverage in lake buffer zones effectively mitigates nutrient and pollutant loads in runoff, thereby safeguarding lake ecosystems. Characteristics of Exogenous regulation denote human interventions in lake management, including periodic adjustments in the amount of recharge water and dredging depths. These interventions directly impact lake ecosystem dynamics such as water levels, water quality, temperature regimes, water habitats, and hydrodynamics. They represent external inputs crucial for maintaining lake water quality while influencing the resilience and stability of lake ecosystems. Evaluation of lake social services includes metrics like annual visitor numbers, public interest levels, and satisfaction ratings, illustrating lakes' capacity to provide valuable social services. In summary, describing urban lake water environments and social functional attributes from perspectives encompassing lake physical structure, shoreline characteristics, water quality, exogenous regulation, and social services provide essential frameworks for guiding lake management, conservation efforts, and sustainable utilization.

### 2.2 Combined Weight Calculation of Indicators Based on Coefficient of Variation and Entropy Weight Method

This study employs a combined weighting approach based on the coefficient of variation method and the entropy weight method to determine weights. The coefficient of variation method not only addresses the equilibrium deficiencies inherent in entropy weighting calculations but also captures the informational content of indicator base data, rendering its weight coefficients more rational than those derived from a single method alone. Within the evaluation index system of this study, weights are calculated directly using the information encapsulated within each indicator via the coefficient of variation method. Indicators exhibiting greater disparities in values, thereby better‐reflecting distinctions among evaluated entities, receive higher scores. The formulas for computing the coefficient of variation and weights are as follows:

(1) $$V_{i}=\frac{\sigma_{i}}{{\bar{x}}_{i}}$$

(2) $$\alpha_{i}=\frac{V_{i}}{\sum_{i=1}^{n}V_{i}}\left(i=1,2,\cdot \cdot \cdot ,n\right)$$

Here, *V_i_* represents the coefficient of variation for the i‐th indicator, σ_*i*_ denotes the standard deviation of the i‐th indicator, ${\bar{x}}_{i}$ signifies the mean of the i‐th indicator, and α_*i*_ denotes the coefficient of variation weight.

The entropy weight method, functioning as an objective weighting approach, reflects latent information embedded within indicators. A lower entropy value corresponds to a higher entropy weight, indicating greater disparity and developmental potential among indicators. Conversely, higher entropy values yield lower entropy weights. The entropy formula is expressed as:

(3) $$S^{*}=\;-\frac{1}{\ln n}\sum_{i\;=\;1}^{n}\frac{x_{ij}}{x^{*}}\ln\frac{x_{ij}}{x^{*}}$$

(4) $$x^{*}=\sum_{i=1}^{n}\left(i=1,2,\cdot \cdot \cdot ,n\right)\left(j=1,2,\cdot \cdot \cdot ,m\right)$$

Here, S* represents the entropy of the j‐th indicator, *x_ij_* signifies the sum of all j‐th indicator values across water bodies.

The entropy weight formula is:

(5) $$\beta_{i}=\frac{1-S^{*}}{m-\sum_{j=1}^{m}S^{*}}$$

Here, β_*i*_ denotes the entropy weight, and *m* signifies the number of indicators.

The combined weighting formula, integrating the coefficient of variation and entropy weight methods, is defined as:

(6) $$W=\lambda \cdot \beta_{i}+\left(1-\lambda \right)\alpha_{i}$$

Here, *W* denotes the geometric mean weighting, β_*i*_ represents the entropy weight, α_*i*_ signifies the weight derived from the coefficient of variation method, and λ denotes the preference coefficient (where λ is set to 0.5 in this study).

This methodological approach integrates robust statistical techniques to comprehensively determine weights for assessing water quality, ensuring a balanced consideration of both variability and information entropy across multiple indicators.

### 2.3 Systematic Health Assessment of Urban Lakes Based on the Synergistic Development Assessment Model (SDAM)

The assessment model of synergetic development integrates a synergistic degree and comprehensive development level to assess the degree of synergy within a system. This model represents an evolution of the capacity coupling model originating from physics, emphasizing mutually beneficial interactions among system components to enhance overall system integrity. The theory of synergetic development serves as a foundational framework for sustainable development, providing theoretical guidance to address the unbalanced nature of regional socio‐economic development and effectively plan various aspects of regional growth. The application of the synergetic development assessment model in evaluating the systemic health of lakes is critical for understanding the interrelationships between water environment quality and the social service functions of lakes. The evaluation system aims to assist practitioners in analyzing and balancing the ecological and social functions of urban lakes more effectively. It underscores the importance of coordinating and developing internal factors influencing lake systems, thereby fostering synergistic development and maximizing overall benefits.

#### 2.3.1 Synergy Index and Synergy Development Assessment Model

The synergistic theory views the research object as a composite system composed of multiple subsystems. These subsystems interact with each other, resulting in overall effects that collectively drive the development and changes of the composite system. In this study, the research focuses on a robust composite system comprising the water environment quality subsystem and the social service function subsystem. Using a synergistic development evaluation model, the objective is to calculate the level of synergistic development between water environment quality and social service functions in different lakes, to understand the relationship between overall development quality and these subsystems. The synergistic development evaluation model, based on the principles of synergistic development theory and system theory, is presented as follows:

(7) $$B\left(t\right)={\left[\frac{f\left(t,x\right)g\left(t,y\right)}{{\left(\frac{f\left(t,x\right)+g\left(t,y\right)}{2}\right)}^{2}}\right]}^{k}$$

In the given equations, *B*(*t*) represents the synergy degree between the two subsystems, *f*(*t*, *x*) and *g*(*t*, *y*) denote the benefit values of each lake, and k is an adjusting coefficient typically ranging from 2 to 5, with a value of 3 in this case. The synergy degree *B*(*t*) ranges from 0 to 1, where a value closer to 1 indicates higher synergy between the subsystems. The formulas for calculating subsystem benefits are as follows:

(8)

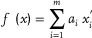



(9)

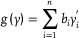

(10) $$C\left(t\right)=\alpha f\left(t,x\right)+\beta g\left(t,y\right)$$

Here, 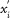 and 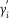 represent the standard values of the corresponding indicators for the subsystems, with i ranging from 1 to n. ai, bi, and ci represent the weights assigned to the indicators. *C*(*t*) represents the comprehensive evaluation value of the system, where α + β = 1. The indicators in the lake system health evaluation system are divided into positive effect indicators and negative effect indicators, and the standard value of each indicator data is calculated. The entropy method is used to calculate the weights of the indicators, which determines the weights by relying on the amount of information conveyed by the indicators to the decision makers, which can reflect the utility of the entropy of the indicators in a more profound way. (Table 1) The system synergy degree (D) is determined by both the synergy degree (*B*(*t*)) and the comprehensive evaluation value *C*(*t*), and it can be calculated as follows:

(11) $$D=\sqrt{B\left(t\right)C\left(t\right)}$$

Here, 0 < D < 1, and a higher value of D indicates a higher degree of synergy between the subsystems. A high synergy degree among the influencing factors within a lake ecosystem reflects similar developments in ecological integrity and social service functionality. However, simultaneous development in both aspects may also indicate relatively low levels of development in either one. To assess the comprehensive development level, the following formula is utilized:

(12) $$w_{i}=\sum_{i=1}^{p}w_{i}\;\;\;w_{j}=\sum_{j=1}^{m}w_{j}$$

(13) $$T\;=w_{i}f\left(t,x\right)+w_{j}g\left(t,y\right)$$

Here, T represents the index of comprehensive development level, reflecting the lake system's health. *w_i_* and *w_j_* represent the weights assigned to the indicators of water environment quality and social service functionality, respectively.

#### 2.3.2 Ranking of Urban Lake System Health and Synergistic Development

Based on China's “Surface Water Environmental Quality Standards” (GB3838‐2002) and references to the grading of lake health evaluation standards and system synergy levels both domestically and internationally, the division of lake system synergy and comprehensive development value into appropriate grades is presented in **Tables** **3** and **4** as follows:

**Table 3:** System Synergy Grades and Classification.

Range	D‐value interval	Grade of synergy	Degree of system synergy
Discoordination decline range	[0.0≈0.1]	1	Severely imbalanced
	[0.1≈0.2]	2	Seriously imbalanced
	[0.2≈0.3]	3	Moderately imbalanced
	[0.3≈0.4]	4	Mildly imbalanced
	[0.4≈0.5]	5	Nearly imbalanced
Synergistic development range	[0.5≈0.6]	6	Barely synergistic
	[0.6≈0.7]	7	Basically synergistic
	[0.7≈0.8]	8	Intermediately synergistic
	[0.8≈0.9]	9	Well synergistic
	[0.9≈1.0]	10	Excellently synergistic

**Table 4:** Classification of comprehensive development level of urban lakes.

Range	T‐value interval	Grade of development	Degree of comprehensive development
Discoordination decline range	[0.0≈0.2]	1	Morbid
	[0.2≈0.4]	2	Unhealthy
Synergistic development range	[0.4≈0.6]	3	Sub‐healthy
	[0.6≈0.8]	4	Healthy
	[0.8≈1.0]	5	Completely healthy

The methodology for assessing lake health, rooted in the coordinated evaluation of water environment quality and social service functionality, establishes a standardized grading system for measuring the vitality of lake ecosystems from dual perspectives: systemic synergy and comprehensive developmental levels. This method enables a thorough evaluation, merging insights into both ecological resilience and the societal roles fulfilled by lake ecosystems. It facilitates the analysis of synergistic interactions and potential for growth. Furthermore, the digitalization of hydrological and social service functionality results enhances the capacity for comparative analysis, shedding light on challenges in the coordinated development of lake ecosystems. This approach aids in pinpointing precise priorities for future development efforts.

### 2.4 Urban Lake Selection and Data Acquisition

Considering the varying developmental stages and environmental conditions of urban lakes, alongside their structural diversity, size, surroundings, and functions, this article limits its focus to the following criteria:

1. Lakes situated in urban plains: Urban lakes in plain regions are characterized by expansive water bodies, gentle terrain, and a relatively autonomous ecosystem within the lake system. These lakes face persistent environmental pressures and complex management challenges.
2. Lakes of comparable size: Size significantly influences the ecological structure and operational dynamics of lakes. Evaluating lakes of similar sizes allows for more precise and comparable assessment indicators, enhancing the understanding of their characteristics and challenges.
3. Lakes located in environments with various urbanization intensities: This implies potential pressures from urban development and disturbances in the vicinity, such as changes in land use and human activities. The chosen evaluation criteria considered the impact of development intensity on lake ecosystems.
4. Lakes demonstrating water quality and substantial social service functionality: The selected assessment criteria comprehensively evaluate water quality and the lakes' contribution to social services, aiming to provide a holistic assessment of their condition and social significance.

**Figure 2:**
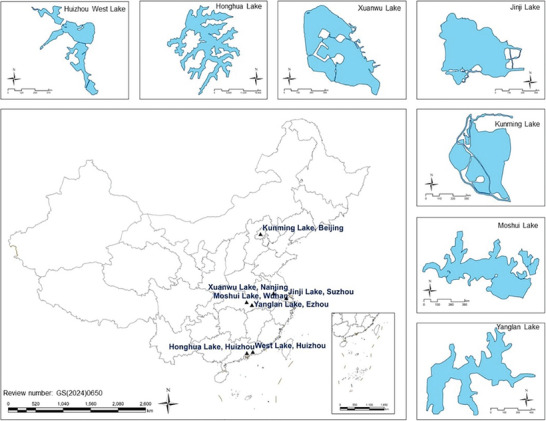
Location and morphological extraction of selected lakes.

Based on these considerations, the specific lakes are detailed in **Table** **5** and **Figure** **2**.

**Table 5:** Basic information on selected lakes.

Selected lakes	Geographical position	Water area [km^−2^]	Urban development (Annual GDP/trillion)
Huizhou West Lake	Huizhou, Guangdong Province	3.13	0.42
Honghua Lake	Huizhou, Guangdong Province	1.62	0.42
Xuanwu Lake	Nanjing, Jiangsu Province	3.78	1.48
Jinji Lake	Suzhou, Jiangsu Province	7.4	2.02
Kunming Lake	Beijing, Capital of China	2.94	3.61
Moshui Lake	Wuhan, Hubei Province	3.45	1.56
Yanglan Lake	Ezhou, Hubei Province	2.88	0.11

The foundational data utilized in this study primarily includes:

1. Satellite‐Based Lake Morphology Extraction and Correction: This technique involves capturing and rectifying lake morphology using a satellite map grabber. The ArcGIS platform is employed to extract water boundaries from satellite images and vectorize them to characterize the physical structural features of the lakes.
2. Compilation of Ecological Environment Bulletins and Publicly Available Lake Statistics: Ecological environment bulletins and related publicly available lake statistics from each city where the lakes are located provide valuable information on the environmental conditions. These data enhance indicators referencing the “Surface Water Environment Quality Standards.”
3. Utilization of Statistical Yearbook Data from Selected Cities: Statistical yearbook data from the cities hosting the lakes represent the urban economic and social development levels. This contextual information supports the evaluation of the lakes by providing insights into the socio‐economic dynamics.

By incorporating these foundational data sources, the study aims to gather comprehensive and reliable information to accurately assess the characteristics and conditions of the lakes.

## 3 Results

### 3.1 Results of Sub‐Indicator Evaluation of Urban Lake Health

Based on remote sensing imagery of the seven selected lakes, lake index data were vectorized and digitized using the ArcGIS platform. Physical structural indicators of the lakes were calculated, statistically analyzed, and integrated using FRAGSTATS software and interpolation methods. Considering the range of indicators within the evaluation system, each indicator value was non‐dimensionalized, and certain indicators underwent interpolation evaluation. Scoring methodology and reference standards adhered to the national “Surface Water Environment Quality Standards” (GB3838‐2002). Linear interpolation determined indicator values between adjacent levels. Weights were assigned according to the lake system health evaluation methodology outlined in Table 2. The standardized indicator values for each lake are presented in **Table** **6** below:

**Table 6:** Standardization of lake system health evaluation indicators.

Indicators	Huizhou West Lake	Honghua Lake	Xuanwu Lake	Jinji Lake	Kunming Lake	Moshui Lake	Yanglan Lake
Lake area	0.39	0.20	0.47	0.93	0.37	0.43	0.36
Lake depth	0.05	0.90	0.05	0.06	0.05	0.05	0.04
Lake shape index	0.34	0.92	0.52	0.42	0.40	0.40	0.53
Shoreline development index	0.75	0.56	0.17	0.10	0.86	0.27	0.17
Lake near‐circularity	0.67	0.68	0.64	0.55	0.75	0.69	0.72
Water quality classification	0.50	0.33	0.67	0.67	0.50	0.83	0.83
COD	0.44	0.32	0.66	0.64	0.42	0.92	0.86
TP	0.06	0.03	0.25	0.44	0.17	0.41	0.40
TN	0.38	0.01	0.69	0.63	0.42	0.71	0.70
DO	0.86	0.93	0.53	0.64	0.86	0.57	0.59
Transparency	0.50	0.33	0.67	0.67	0.50	0.83	0.83
Shoreland green space to lake area ratio	0.40	0.91	0.07	0.11	0.19	0.10	0.20
Percentage of vegetation cover in the 60‐meter buffer zone	0.34	0.40	0.42	0.45	0.45	0.43	0.43
Amount of recharge water	0.02	0.00	0.24	0.97	0.01	0.00	0.03
Dredging depth	0.86	0.00	0.71	0.71	0.00	0.63	0.29
Number of tourist Visits	0.51	0.13	0.98	0.33	0.21	0.01	0.00
Concerned about the heat	0.22	0.08	0.99	0.82	0.31	0.09	0.02
Public satisfaction index	0.65	0.55	0.75	0.80	0.65	0.45	0.55

### 3.2 Results of Synergistic Assessment of Water Ecological Quality and Social Service Functions of Urban Lakes

In accordance with the established collaborative development evaluation model, the results obtained for the water environment quality and social service functionality composite system of seven lakes, as well as the synergy status and comprehensive development level of their two subsystems, are presented in **Tables** **7** and **8**. The city's GDP can to some extent serve as an indicator of urban development level and governance capability. From the provided tables, it is evident that the synergy between lake water environment quality and service functionality exhibits significant variations across cities with different levels of development. Lakes located in cities with lower levels of development, such as Moshui Lake and Yanglan Lake, demonstrate correspondingly lower water environment quality and service functionality, portraying an overall state of sub‐health and contraction in their developmental trajectory. On the other hand, lakes situated in mid‐level developmental cities like Huizhou West Lake and Honghua Lake exhibit an appropriate degree of development in terms of social service functionality, while also maintaining a commendable water environment quality. It is this harmonious interplay between the two factors that contributes to the healthy development of the lake systems. In contrast, cities with higher levels of development such as Nanjing and Hangzhou, where Xuanwu Lake and Jinji Lake are located, showcase lakes with relatively high levels of social service functionality but comparatively weaker water environment quality. Despite the governance stemming from large‐scale water replenishment, dredging, and other exogenous forces employed to maintain the water ecological systems of the lakes, the water quality indicators still reflect a sub‐healthy level, resulting in a degraded state of the lake ecosystems. Nevertheless, lakes found in cities with higher levels of development, such as Kunming Lake in Beijing, present a coherent development pattern where ecological environment quality and service functionality work in synergy, thereby portraying a healthy and comprehensive state of advancement. These findings underscore the fact that the intensity of engineered lake management is not always consistent with water environmental quality, emphasizing that the attainment of lake health necessitates a systematic approach to water environment governance. Engaging in the process of restoring lake ecosystems serves as a sustainable means of enhancing their overall health status.

**Table 7:** Results of lake system synergy assessment.

Lake	Synergy Index	System synergy index	Degree of synergy development index
Huizhou West Lake	0.94	0.67	Basically synergistic
Honghua Lake	0.82	0.76	Intermediately synergistic
Xuanwu Lake	0.50	0.49	Nearly imbalanced
Jinji Lake	0.31	0.33	Mildly imbalanced
Kunming Lake	0.95	0.83	Well synergistic
Moshui Lake	0.95	0.13	Seriously imbalanced
Yanglan Lake	0.92	0.31	Mildly imbalanced

**Table 8:** Results of the level of comprehensive development of lakes.

Lake	w_i_	w_j_	T	Degree of comprehensive development
Huizhou West Lake	0.4883	0.5138	0.5002	Healthy
Honghua Lake	0.8023	0.3085	0.5722	Healthy
Xuanwu Lake	0.2950	0.8885	0.5716	Sub‐healthy
Jinji Lake	0.2653	0.6412	0.4405	Sub‐healthy
Kunming Lake	0.6188	0.6001	0.6101	Healthy
Moshui Lake	0.2542	0.0318	0.1506	Morbid
Yanglan Lake	0.3180	0.0909	0.2122	Unhealthy

## 4 Discussion

### 4.1 Feedback Between Lake System Health and Level of Urban Development

The urban lake is a complex system, necessitating a thorough comprehension of its holistic nature to accurately gauge its health. Research employing the SDAM has illuminated two distinct feedback loops between lake system vitality and urban development levels:

On one hand, urban development significantly influences the overall health of lakes. In China, the urbanization process and exploitation of lake resources have continually disrupted the spatial integrity of lakes through watershed construction and direct lakefront activities. However, achieving synergy between urban planning and the lakes' self‐organizing capacity remains a formidable challenge. For example, in urban lakes such as Moshui Lake, water quality deteriorates as urban development progresses, leading to a decline in the lake system's health. Moreover, despite their potential, the full spectrum of their social service functions remains underutilized, resulting in a contracted state of lake system health. In cities with more advanced development, lake resources are fully tapped, and social service functions are enhanced. Simultaneously, the promotion of ecological civilization concepts has bolstered efforts in ecological conservation. Urban lakes like Xuanwu Lake and Jinji Lake serve as crucial recreational areas within cities, with proactive human interventions to preserve their ecological integrity.^[^ ^46^ ^]^ However, assessments of synergistic impacts reveal that while social service functions may improve, there is a concurrent decline in water quality, contributing to an overall suboptimal state of the lakes. This underscores the inherent tension between urbanization and ecological preservation.

On the other hand, the enhancement of urban construction quality and governance capacity promotes the healthy development of urban lake water environments. Currently, there exists a paradox: as urbanization continues to progress, people's living demands are increasing. Creating conditions to meet these requirements inevitably leads to high‐level regional development, which, in turn, negatively impacts the urban environment. Numerous research findings confirm that urban development results in fragmentation, shrinkage, and functional decline of urban green spaces and water bodies over time. At first glance, it may seem that the economic and social development of cities conflicts with the preservation of urban ecological environments. However, while urbanization does pose certain threats to the ecological environment, various safeguards have been implemented as urban development progresses and construction quality improves. For instance, in the present study, Huizhou West Lake and Honghua Lake exhibited a relatively healthy state due to being scenic spots and protected water sources within the city, benefiting from greater conservation efforts. Beijing's Kunming Lake displayed a synergistic development of water environment quality, service functions, and comprehensive health status. This achievement can be attributed to the strong protection and governance measures implemented under high urban governance capacity. A separate study also provides ample evidence supporting this claim. Researchers investigated the spatiotemporal variations of Chaohu Lake, from 2011 to 2020, evaluating the relationship between water quality and socio‐economic factors. The results indicated that the western region of the Chaohu Lake basin experienced a concentration of residential areas, with significant industrial and domestic wastewater contributing to water pollution in the western tributaries. TP and TN were identified as the major pollution parameters for Chaohu Lake. However, due to human intervention, the pollution situation has gradually improved. From 2011 to 2020, water quality improved by 23% to 35% in the western region and by 7% to 14% in the eastern region. This demonstrates that urbanization does not necessarily imply continuous harm to the urban ecological environment. Instead, a shift in development concepts and an increase in governance capacity have contributed to enhancing the health level of urban lakes.

### 4.2 Synergizing Water Environment and Social Service Functions for Urban Lake Health

From a perspective of comprehensive and sustainable development, enhancing lake health systemically requires more than relying solely on engineering interventions for water environment management. Taking Xuanwu Lake and Jinji Lake as examples, despite maintaining water quality through extensive external water flushing and regular dredging to ensure aesthetic appeal and provide social benefits, their synergy assessments reveal a state of “near imbalance” and “mild imbalance.” In terms of comprehensive development, they exhibit a “sub‐healthy” status. In contrast, Honghua Lake in Huizhou experiences fewer external disturbances, showing “intermediate synergistic” and achieving a “healthy” status in comprehensive development. Honghua Lake maintains superior water quality primarily due to the healthy functioning of its hydrological processes, which ensure sustained self‐regulation of the lake.

Indicators such as “recharge water quantity” and “dredging depth” should be considered as bidirectional factors. The health of a lake is intricately linked to its hydrological processes. Frequent external disturbances could disrupt a lake's ability to self‐regulate, leading to health deterioration. To comprehensively enhance lake health, it is crucial to adopt a broader spatiotemporal perspective, such as restoring the hydrological processes of the lake's watershed and allowing natural processes to play a more significant role. This approach enhances the overall development of the lake system and strengthens its adaptive capacity in response to both human activities and natural influences.

### 4.3 Analysis of System Health in the Case of Xuanwu Lake

The study delves deeper into examining the lack of synergy between the water environmental quality and social service functionalities at Xuanwu Lake, while also analyzing the bidirectional impacts of external regulatory measures such as water recharging and dredging. Xuanwu Lake presents characteristics typical of a plain lake, with a central dish‐shaped depression. Urban terrain features dictate its watershed sources, primarily encompassing the northern slopes of Zhong Mountain, the southern slopes of Mufu Mountain, and the northeastern watershed areas. However, the development of construction land has altered the lake's hydrological boundaries, impeding the natural inflow of surface water into Xuanwu Lake and severely restricting hydrological processes and ecological functions. For instance, runoff from the northern slopes of Purple Mountain originally flowed westward into Xuanwu Lake. The construction of the Junmin Friendship Reservoir regulates and diverts water from the northern slopes, channeling it across the watershed to irrigate areas like the State Guesthouse, Zhongshan Golf Course, and villa districts. Various anthropogenic factors along the runoff paths of Xuanwu Lake have significantly intercepted or obstructed surface runoff processes, resulting in imbalances in the surface water environment of the watershed.

Given the increasing recreational significance of Xuanwu Lake, efforts to ensure it meets social service demands include environmental management initiatives such as water diversion, dredging, and pollution interception. Currently, over 200 000 tons of Yangtze River water are transferred daily across watersheds to flush the lake, with an annual replenishment volume exceeding 70 million tons, extremely costly to maintain. While water diversion projects have contributed to some improvement in lake water quality, the introduction of significant volumes of Yangtze River water has maintained Xuanwu Lake in a mildly to moderately eutrophic “sub‐healthy” state. On one hand, frequent and substantial water diversions have disrupted the ecological balance of the lake and its aquatic ecosystems. On the other hand, despite sedimentation and purification of Yangtze River water sources, high levels of total nitrogen, ammonia nitrogen, and total phosphorus persist, meeting riverine standards but falling short of those required for a lake environment, thereby failing to fundamentally resolve water quality issues. Furthermore, Xuanwu Lake utilizes water intake dredging to remove substantial quantities of surface sediment and mitigate sediment pollution. However, lake sediment contains significant amounts of nitrogen, phosphorus, and heavy metals, and continued disturbances from dredging and water diversion projects further exacerbate the continuous release of nutrients, impacting the germination and growth of aquatic plant reproductive bodies and disrupting the stability of the biological chain cycle. While dredging projects may yield short‐term improvements in water quality, they do not represent sustainable measures for addressing Xuanwu Lake's eutrophication in the long term. The development of aquatic organisms necessitates suitable external water flow conditions, posing heightened requirements for water diversion from an ecological hydraulics perspective. Overall, Xuanwu Lake presently relies on environmental management initiatives to uphold its existing aquatic landscape, exhibiting relatively low self‐sustaining and disturbance resistance capabilities.

### 4.4 Systematic Strategies for Synergistic Development of Xuanwu Lake

The study explores strategies aimed at enhancing the holistic health of Xuanwu Lake through a collaborative development perspective. Historically, Xuanwu Lake's natural body has been sustained by effective water collection and drainage relationships. However, disruptions in surface hydrological processes have significantly impacted the lake's environmental health. Therefore, the governance of Xuanwu Lake's water environment necessitates not only the restoration of the lake itself but also the rehabilitation of hydrological processes within its small watershed and sustainable water sources.

By analyzing the urban terrain features that define Xuanwu Lake's catchment area and runoff paths, the annual average runoff within this scope is estimated at ≈21.6 million cubic meters. Maximizing the collection of runoff within the small watershed, existing water bodies are prioritized. Through methods such as communication and dredging of surface water systems, efforts are directed toward restoring the original hydrological processes to channel surface water into the lake. Furthermore, conflicts between natural hydrological processes and land development within urban lake watersheds are common. Depending on the actual conditions of urban development, selective restoration of water systems or bodies in feasible locations is pursued. For instance, restoring surface runoff channels on the western slopes of Purple Mountain and potential runoff pathways from Mufu Mountain to the north of Hong Mountain could respectively restore annual rainfall volumes of 4.32 million and 5.4 million cubic meters. Reconstructing the small watershed network greatly enhances the hydrological processes of Xuanwu Lake's watershed environment. By simulating natural processes and minimizing energy consumption, fundamental improvements and restoration of Xuanwu Lake's hydrological processes are pursued.

Second, restoring hydrological processes improves urban land's permeability and retention of rainwater. In cases where natural hydrological processes cannot be fully restored, strategies such as nearby drainage diversion, promoting infiltration, and integrating sponge city measures are implemented to enhance rainwater retention capacity in the small watershed. Ecological construction serves as essential interfaces for infiltration, runoff, and other natural hydrological processes, maintaining stable hydrological cycles to increase soil and near‐surface water content, effectively supplementing lake water.

Additionally, the layout of urban stormwater pipes typically adheres to original topographic elevations, and organized stormwater discharge retains quasi‐natural effects. The topographic features of basin depressions collect urban stormwater runoff effectively. Therefore, stormwater discharge should utilize vertical conditions fully to organize stormwater entry into the lake. For example, aggregating stormwater pipes from small watersheds, combined with culvert transport and surface runoff into eastern channels like Purple Mountain Gully and Tangjiashan Gully, involves necessary purification measures based on water quality conditions, gradually reducing excessive dependence on artificial water supplementation, exchange, and water level regulation.

Based on current urban development and construction statuses, effective coordination of engineering technologies and ecological measures through methods such as restoring watershed runoff and infiltration processes, combined with existing grey infrastructure, significantly increases surface runoff storage space. This approach not only supplements lake water sources but also restores existing hydrological processes within the watershed to allow nature to play its role maximally. This comprehensive approach contributes to the holistic development of lake system health, enhancing its capacity for self‐sustenance and regulation.

## 5 Conclusion

Urban lakes are intricate ecological systems where the condition of their water environments emerges from a complex interplay of numerous factors. The water environment assessment of urban lakes emphasizes the specific spatiotemporal manifestation, alongside their periodic exogenous regulations characteristics. Evaluation is conducted across four dimensions: physical structure, water quality, shoreline characteristics, and exogenous regulation, to provide a comprehensive understanding of the structural, functional, and stability aspects of urban lakes, thereby offering a foundation for lake management and conservation. It facilitates ongoing enhancement and sustainable utilization of urban lake ecosystems.

In contrast to general evaluations of spatial environments such as “ecological, social, economic” integration, this study employs a SDAM. It focuses on two subsystems of critical importance: water environment quality and societal functions. By examining their synergistic relationships, deeper insights into the developmental conditions of urban lakes are gained. The established assessment methodology facilitates systematic comparative evaluations of health levels across multiple lakes and longitudinal assessments of health levels within a single lake. Notably, it excels in computational efficiency and broad applicability. Comparative analysis of subsystem synergy and comprehensive development levels among lakes illuminates strategic priorities for each lake's development and scientific management.

## Conflict of Interest

The authors declare no conflict of interest.

## Data Availability

The data that support the findings of this study are openly available in Geospatial Data Cloud at https://www.gscloud.cn, reference number 32118.
